# Surdité brutale révélant un syndrome de Vogt-Koyanagi-Harada

**DOI:** 10.11604/pamj.2015.22.103.8006

**Published:** 2015-10-06

**Authors:** Madiha Mahfoudhi, Khaled Khamassi

**Affiliations:** 1 Service de Médecine Interne A, Hôpital Charles Nicolle, Tunis, Tunisie; 2 Service ORL, Hôpital Charles Nicolle, Tunis, Tunisie

**Keywords:** Syndrome de Vogt-Koyanagi-Harada, surdité, méningite, Vogt-Koyanagi-Harada syndrome, deafness, meningitis

## Image en medicine

Le syndrome de Vogt-Koyanagi-Harada est une affection rare, caractérisée par l'association de manifestations oculaires, méningées, auditives et cutanées. L'atteinte cochléo-vestibulaire se voit dans 33 à 75% des cas. La surdité est généralement d’évolution progressive, exceptionnellement brutale. La sévérité de cette atteinte est variable. Le pronostic est amélioré par un diagnostic précoce et une prise en charge thérapeutique adéquate basée sur une corticothérapie prolongée parfois associée à des immunosuppresseurs. Patiente âgée de 43 ans, présentant un vitiligo qui remontait à 3 ans, a consulté pour une surdité brutale de l'oreille droite, un vertige rotatoire, des acouphènes, des vomissements, des céphalées occipitales, une baisse de l'acuité visuelle et une photophobie. L'examen physique a objectivé un nystagmus horizonto-rotatoire battant à gauche et un examen neurologique normal. L’étude du liquide céphalo-rachidien a montré une méningite lymphocytaire aseptique. L'examen ophtalmologique a révélé une pan-uvéite granulomateuse bilatérale et un aspect dépigmenté de la rétine au fond d'oeil. Plusieurs diagnostics ont été suspectés en particulier un lymphome et une sarcoïdose. L'otoscopie était normale. L'audiométrie tonale a montré une subcophose droite et une audition normale à gauche. L’épreuve calorique a trouvé une hyporéflexie droite. L'IRM cérébrale, des conduits auditifs et des rochers était normale. Le diagnostic d'une poussée aigue de maladie de Vogt-Koyanagi-Harada a été retenu. Le traitement s'est basé sur des corticoïdes, des anti-vertigineux, un vasodilatateur et des topiques oculaires. L’évolution était marquée par une régression complète de l'atteinte oculaire et une récupération partielle de l'audition.

**Figure 1:**
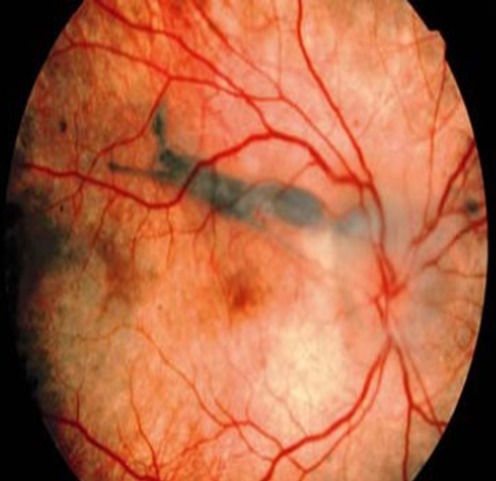
Dépigmentation rétinienne

